# Incidence of cancer in the area around Amsterdam Airport Schiphol in 1988–2003: a population-based ecological study

**DOI:** 10.1186/1471-2458-5-127

**Published:** 2005-12-06

**Authors:** Otto Visser, Joop H van Wijnen, Flora E van Leeuwen

**Affiliations:** 1 Comprehensive Cancer Centre Amsterdam, POBox 9236, 1006 AE Amsterdam, The Netherlands; 2 Municipal Health Service Amsterdam, Environmental Medicine, POBox 20244, 1000 HE Amsterdam, The Netherlands; 3 Netherlands Cancer Institute, Dept of Epidemiology, Plesmanlaan 121, 1066 CX Amsterdam, The Netherlands

## Abstract

**Background:**

Amsterdam Airport Schiphol is a major source of complaints about aircraft noise, safety risks and concerns about long term adverse health effects, including cancer. We investigated whether residents of the area around Schiphol are at higher risk of developing cancer than the general Dutch population.

**Methods:**

In a population-based study using the regional cancer registry, we estimated the cancer incidence during 1988–2003 in residents of the area surrounding Schiphol. We defined a study area based on aircraft noise contours and 4-digit postal code areas, since historical data on ambient air pollution were not available and recent emission data did not differ from the background urban air quality.

**Results:**

In residents of the study area 13 207 cancer cases were diagnosed, which was close to the expected number, using national incidence rates as a reference (standardized incidence ratio [SIR] 1.02). We found a statistically significantly increased incidence of hematological malignancies (SIR 1.12, 95% confidence interval [CI]: 1.05, 1.19), mainly due to high rates for non-Hodgkin lymphoma (SIR 1.22, 95% CI: 1.12, 1.33) and acute lymphoblastic leukemia (SIR 1.34, 95% CI: 0.95, 1.83). The incidence of cancer of the respiratory system was statistically significantly decreased (SIR 0.94, 95% CI: 0.90, 0.99), due to the low rate in males (SIR 0.89). In the core zone of the study area, cancer incidence was slightly higher than in the remaining ring zone (rate ratio of the core zone compared to the ring zone 1.05, 95% CI 1.01, 1.10). This was caused by the higher incidence of cancer of the respiratory system, prostate and the female genital organs in the core zone in comparison to the ring zone.

**Conclusion:**

The overall cancer incidence in the Schiphol area was similar to the national incidence. The moderately increased risk of hematological malignancies could not be explained by higher levels of ambient air pollution in the Schiphol area. This observation warrants further research, for example in a study with focus on substances in urban ambient air pollution, as similar findings were observed in Greater Amsterdam.

## Background

Amsterdam Airport Schiphol is one of the main airports of Europe. The airport is a major source of complaints about aircraft noise, noise related adverse health effects and – especially since the crash of an airplane in a suburb of Amsterdam on October 4^th ^1992 – about safety risks. A longstanding subject of concern of the surrounding population is the exposure to aviation fuels and their combustion products and an alleged increase of cancer risk. Particularly in warm summers the smell of aviation fuels can be distinguished outside the airport grounds. Aircraft emissions vary with the engine type, the engine load and the kind of fuel. Combustion of aviation fuels results in CO_2_, CO, C_e_, NO_x_, particles, and a great number of other organic compounds, among which a number of carcinogens [1]. Among the emitted polycyclic aromatic hydrocarbons no compound characteristic for aircraft engines has been detected so far.

A committee of the Health Council of The Netherlands recently reviewed the data on the health impact of large airports [2]. It was concluded that, generally, integrated health assessments are not available. In the last 30 years, several adverse health effects in relation to exposure to aircraft noise have been the subject of study, such as the use of tranquillizers, the prevalence of bronchitis and cardiovascular disease as well as child stress responses and cognition [3-6]. However, little information is available in the international literature on cancer risk in relation to airports.

In the late 1980s, mortality due to cancer in the community of Haarlemmermeer, which hosts Schiphol, was investigated by the Municipal Health Service of Amsterdam on request of the general practitioners in the area [7]. The total cancer mortality and the lung cancer mortality in Haarlemmermeer during 1981–86 did not differ statistically significantly from the cancer mortality in the two standard populations that were used. The mortality due to non-Hodgkin lymphoma (NHL) was statistically significantly increased, but conclusions as to the cause of the excess mortality were not possible.

In the 1990s, we carried out a first study on the incidence of cancer in the vicinity of Schiphol, as part of the health surveillance of the resident population of the Schiphol area [8]. During 1988–1993, the incidence of cancer in the area around Schiphol was close to the national average. The differences in incidence of certain types of cancer in comparison to the national average, as well as those between two study areas characterized by different levels of increased aircraft noise, were considered to be most likely due to differences in life style, such as smoking. In order to investigate whether cancer risk of the resident population of the Schiphol area (in comparison to the national average) changed since 1988–1993, we continued monitoring cancer incidence and we report here on the second, much larger population-based study of the cancer incidence around Schiphol.

## Methods

### Definition of the study population and the study area

**Figure 1:**
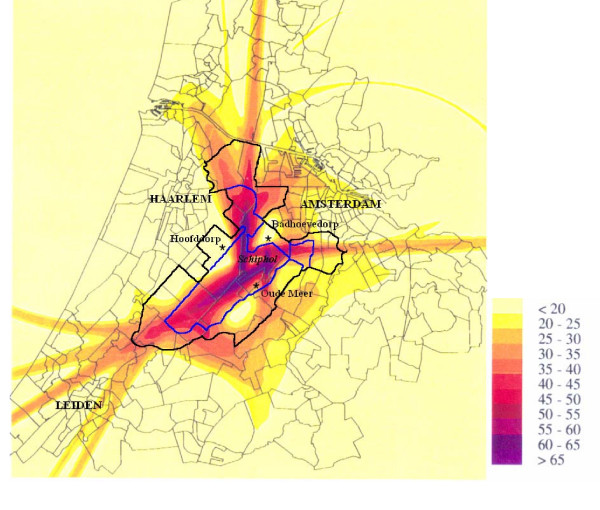
Noise exposure (in Kosten-units) in the Schiphol area in 1991. The area surrounded by the blue line indicates the core zone, the black line includes the total study area. The location of the three air quality monitoring stations are indicated by asterisks (*).

When we designed our first study, relevant exposure data on the ambient air quality around Schiphol airport were lacking and we could not define a study population exposed to increased ambient levels of aircraft emissions. The airport itself has no permanent residents and the most heavily exposed population – the airport personnel and the travelers – cannot be defined geographically. Therefore, we defined our study population as the population most heavily exposed to increased levels of aircraft noise. Since 1994, the ambient air quality outside Schiphol has been monitored and no differences with the background urban air quality have been reported for the compounds that were measured [9]. Table 1 summarizes the results of the three monitoring locations in the Schiphol area. However, it is possible that exposure to aircraft emissions has been greater in the past when aircraft engines used to be technologically and ecologically less advanced. Also, we cannot exclude that certain carcinogenic compounds specific to aviation combustion have not been monitored. Since most cancers have a long induction period and the noise contours are thought to reflect best the historical exposure of the surrounding population to aircraft emissions, we continued to use the levels of aircraft noise to define our study area. The aircraft noise levels of 1991 were available as so-called Kosten-units (Ku) [10]. We used the 35 Ku contour and extended the area with about 2 km outside the 35 Ku contour (figure 1). This total area (surrounded by the solid black line in figure 1) was redefined as 4-digit postal code areas (postal code areas surrounded by grey lines in figure 1). The four airstrips of the airport are easily recognized by noise levels over 50 Ku. We also defined a core zone for the 4-digit postal code areas within the 45 Ku contour (the area bordered by the blue line in figure 1), although we do not have empirical data showing that this zone corresponds to a zone with increased levels of ambient air pollution. The remaining study area surrounding the core zone we designated as 'ring zone'. The location of the three air quality monitoring stations (Badhoevedorp, Hoofddorp, Oude Meer) is also indicated in figure 1. The total study area with a population of 177 000 on 31 December 2003 comprised (parts of) five municipalities (table 2). Table 2 also includes figures on per capita income as approximation for socio-economic status.

**Table 1:** Summary of the results in μg/m^3 ^(except benzo(a)pyrene: ng/m^3^) of the air quality monitoring system of the Schiphol area in 2002

*Pollutant*	*Unit*	*Limit*	*Location of monitoring station*		
			
			*Badhoevedorp*	*Oude Meer*	*Hoofddorp*
NO_2_	year average	40^a^	38	38	31
	maximum	200^b^	163	544(1x > 200)	124
CO	P98 (8 hours)	9000	112	100	88
	P99,9	40000	134	165	160
O_3_	maximum	240^c^	185	174	266 (2x > 240)
PM_10_	average (year)	40^d,e^	26	24	28
	maximum (24 hours)	50^d,f^	81 (13x > 50)	81 (8x > 50)	132 (22x > 50)
Benzene	year average	10	1.4	1.1	0.7
Black smoke	P98 (24 hours)	90^g^	42	48	34
Benzo(a)pyrene	year average	1	0.14		

^a ^as of 1-1-2010

^b ^exceeding of the limit no more than 18 times per annum

^c ^exceeding of the limit not more than 48 hours

^d ^including factor 1.3

^e ^as of 1-1-2005

^f ^exceeding of the limit no more than 35 times per annum

^g ^the limit expired in July 2001

PM_10 _= particulate matter <10 μm

**Table 2:** Some characteristics of the Schiphol study area

*Zone and municipality*	*Postal codes*	*Inhabitants*		Per capita* income (1998)
			
		*1-1-1988*	*31-12-2003*	
**Core zone**		**30 590**	**31 850**	**€ 10 900**†
Haarlemmermeer	1161	8300	7820	€ 10 700
	1175, 1435-8, 2143	6880	6015	€ 11 000
	2132	7815	10 965	€ 11 000
	2153	3520	3310	€ 10 500
Amstelveen	1182	4075	3740	€ 11 500
**Ring zone**		**131 210**	**144870**	**€ 11 800**†
Haarlemmermeer	1171	10 750	11 770	€ 13 200
	2131	10 205	11 030	€ 11 400
	2151-2	11 925	22 260	€ 11 100
	2154-8, 2165	5585	5940	€ 10 300
Amstelveen	1181, 1183	31 145	30 385	€ 12 400
Amsterdam	1067, 1081-3	34 960	35 080	€ 14 500
Aalsmeer	1431-3	21 740	22 870	€ 11 300
Haarlemmerliede & Spaarnwoude	1165, 2064-5	4900	5535	€ 10 800
**Total study area**		**161 800**	**176 720**	**€ 11 700**†

* the national average in 1998 was € 10 000

† weighed average, rounded to € 100

### Population data

Annual population data covering the period 1995–2003 according to 4-digit postal code, 5-year age groups and sex, were available for all municipalities from Statistics Netherlands. For the period 1988–1994 we used data from the municipal administrations.

### Cancer registry data

The Amsterdam Cancer Registry (ACR) is a regional, population-based cancer registry with complete regional coverage since 1988. The ACR is part of the nation-wide Netherlands Cancer Registry (NCR) [11]. Completeness of the NCR is estimated to be over 95%. The information is extracted from the medical records by registration clerks. Apart from demographic data, data are collected on tumor site, morphological classification (according to the International Classification of Diseases for Oncology [ICD-O], versions 1 and 2), stage of the tumor and treatment of the patients. The third version of the ICD-O was introduced in the NCR for cases diagnosed as of January 2001. Cases diagnosed in a hospital outside the ACR region but with residence in the ACR region are routinely obtained from the national registry and included in our regional registry. Consequently, these cases could be included in the study.

We selected from the registry all cancer cases in the period 1988–2003 with residence in the area around Schiphol airport at the date of diagnosis. We stratified the cases according to type of cancer (or group of cancers), area of residence (core zone or the ring zone), 5-year age group and sex.

### Statistical methods

In our analysis, the incidence of cancer in the national population of the Netherlands served as the reference entity. The expected numbers of cancer (E) for the Schiphol area were calculated for three periods (1988–1993, 1994–1998 and 1999–2003), based on the population data of the Schiphol area (according to 5-year age category and sex) and the 5-year age category and sex-specific cancer incidence rates from the NCR. For the period 1988–1993 we used the average incidence rates of the NCR covering the period 1989–1993 [12], because data for 1988 were not available from the NCR. For the periods 1994–1998 and 1999–2003 we used NCR-data covering 1994–1998 and 1999–2003, respectively [13]. The expected numbers were compared with the observed numbers (O) and standardized incidence ratios (SIRs) were calculated as the ratio between the observed and expected numbers. Exact 95%-confidence intervals (CI) based on the Poisson distribution of O were calculated using STATA 7.0 (STATA Corporation. College Station, Texas, USA). Rate ratios (RR) for the core zone were calculated by dividing the standardized incidence ratio of the core zone by the rate of the ring zone. Ninety five percent CIs of RRs were calculated assuming a log-normal distribution [14].

## Results

In 1988–2003, a total of 13 207 cancers (6 739 in males, 6 468 in females) were diagnosed among residents of the Schiphol area (table 3), which included 2 352 cases among residents of the core zone.

**Table 3:** Observed (O) and expected (E) number of cancers in subjects with residence in the Schiphol area according to site, gender and period of diagnosis, 1988–2003

*cancer site (ICD-10 code) and gender*	*Total period (1988–2003)*				*1988–1993*				*1994–1998*				*1999–2003*			
				
	O	E	SIR	95% CI	O	E	SIR	95% CI	O	E	SIR	95% CI	O	E	SIR	95% CI
All malignancies (C00–C96)	13207	13007.9	**1.02**	1.00, 1.03	4624	4538.6	**1.02**	0.99, 1.05	4220	4125.1	**1.02**	0.99, 1.05	4363	4344.3	**1.00**	0.97, 1.03
adult males	6697	6713.7	**1.00**	0.97, 1.02	2402	2363.5	**1.02**	0.98, 1.06	2145	2150.3	**1.00**	0.96, 1.04	2150	2199.9	**0.98**	0.94, 1.02
adult females	6436	6235.3	1.03*	1.01, 1.06	2190	2154.3	**1.02**	0.97, 1.06	2057	1956.5	1.05*	1.01, 1.10	2189	2124.5	**1.03**	0.99, 1.07
children <15	74	58.9	**1.26**	0.99, 1.58	32	20.7	1.54*	1.06, 2.18	18	18.3	**0.98**	0.58, 1.55	24	19.9	**1.21**	0.77, 1.79
Head & neck (C00–C14)	282	272.9	**1.03**	0.92, 1.16	93	93.7	**0.99**	0.80, 1.22	91	85.9	**1.06**	0.85, 1.30	98	93.3	**1.05**	0.85, 1.28
males	162	176.9	**0.92**	0.78, 1.07	53	62.8	**0.84**	0.63, 1.10	56	55.6	**1.01**	0.76, 1.31	53	58.4	**0.91**	0.68, 1.19
females	120	96.0	1.25*	1.04, 1.49	40	30.9	**1.29**	0.92, 1.76	35	30.3	**1.16**	0.80, 1.61	45	34.8	**1.29**	0.94, 1.73
Gastrointestinal tract (C15–C26)	2889	2936.0	**0.98**	0.95, 1.02	1034	1049.3	**0.99**	0.93, 1.05	899	920.4	**0.98**	0.91, 1.04	956	966.3	**0.99**	0.93, 1.05
males	1494	1538.0	**0.97**	0.92, 1.02	517	541.6	**0.95**	0.87, 1.04	470	482.3	**0.97**	0.89, 1.07	507	514.1	**0.99**	0.90, 1.08
females	1395	1398.1	**1.00**	0.95, 1.05	517	507.7	**1.02**	0.93, 1.11	429	438.1	**0.98**	0.89, 1.08	449	452.3	**0.99**	0.90, 1.09
Respiratory system (C30–C34)	1862	1975.1	0.94*	0.90, 0.99	749	752.6	**1.00**	0.93, 1.07	542	627.1	**0.86**†	0.79, 0.94	571	595.4	**0.96**	0.88, 1.04
males	1378	1548.0	**0.89**†	0.84, 0.94	604	626.3	**0.96**	0.89, 1.01	386	492.7	**0.78**†	0.71, 0.87	388	429.0	**0.90**	0.82, 1.00
females	484	427.1	1.13*	1.03, 1.24	145	126.3	**1.15**	0.97, 1.35	156	134.5	**1.16**	0.98, 1.36	183	166.4	**1.10**	0.95, 1.27
Breast (C50)	2087	1983.6	**1.05**	0.99, 1.11	679	676.4	**1.00**	0.93, 1.08	678	620.3	1.09*	1.01, 1.18	730	686.9	**1.06**	0.99, 1.14
Female genital organs (C51–C58)	710	730.7	**0.97**	0.90, 1.05	252	274.9	**0.92**	0.81, 1.04	237	232.9	**1.02**	0.89, 1.16	221	222.9	**0.99**	0.87, 1.13
Prostate (C61)	1291	1230.9	**1.05**	0.99, 1.11	382	364.6	**1.05**	0.95, 1.16	470	421.2	1.12*	1.02, 1.22	439	445.2	**0.99**	0.90, 1.08
Bladder & other urinary tract (C65–C68)	543	517.2	**1.05**	0.96, 1.14	211	183.9	**1.15**	1.00, 1.31	172	163.7	**1.05**	0.90, 1.22	160	169.7	**0.94**	0.80, 1.10
males	425	390.0	**1.09**	0.99, 1.20	173	139.7	**1.24**†	1.06, 1.44	129	123.6	**1.04**	0.87, 1.24	123	126.7	**0.97**	0.81, 1.16
females	118	127.3	**0.93**	0.77, 1.11	38	44.2	**0.86**	0.54, 1.29	43	40.1	**1.07**	0.78, 1.44	37	43.0	**0.86**	0.61, 1.19
Hematological malignancies (C81–C96)	1044	935.2	**1.12**†	1.05, 1.19	367	328.3	1.12*	1.01, 1.24	334	291.2	1.15*	1.03, 1.28	343	315.8	**1.09**	0.97, 1.21
males	598	507.6	**1.18**†	1.09, 1.28	210	177.3	1.18*	1.03, 1.36	184	157.8	**1.17**	1.00, 1.35	204	172.6	1.18*	1.03, 1.36
females	446	427.6	**1.04**	0.95, 1.14	157	151.0	**1.04**	0.88, 1.22	150	133.4	**1.12**	0.95, 1.32	139	143.2	**0.97**	0.82, 1.15
Hodgkin lymphoma	48	61.2	**0.78**	0.58, 1.04	19	23.6	**0.81**	0.48, 1.26	12	17.7	**0.68**	0.35, 1.18	17	19.9	**0.85**	0.50, 1.37
non-Hodgkin lymphoma	516	423.5	**1.22**†	1.12, 1.33	176	150.0	1.17*	1.01, 1.36	181	133.5	**1.36**†	1.17, 1.57	159	140.0	**1.14**	0.97, 1.33
plasma cell tumors	169	156.1	**1.08**	0.93, 1.26	59	55.6	**1.06**	0.81, 1.37	56	51.3	**1.09**	0.82, 1.42	54	49.3	**1.10**	0.82, 1.43
acute lymphoblastic leukemia	39	29.1	**1.34**	0.95, 1.83	17	10.2	**1.67**	0.97, 2.67	8	8.8	**0.91**	0.39, 1.79	14	10.2	**1.38**	0.75, 2.30
chronic lymphocytic leukemia	92	101.2	**0.91**	0.73, 1.11	33	35.0	**0.94**	0.65, 1.32	29	35.2	**0.82**	0.55, 1.18	30	31.0	**0.97**	0.65, 1.38
acute myeloid leukemia	109	97.8	**1.11**	0.92, 1.34	40	33.9	**1.18**	0.84, 1.61	33	31.4	**1.05**	0.72, 1.48	36	32.4	**1.11**	0.78, 1.54
other	71	66.2	**1.07**	0.84, 1.35	23	20.1	**1.15**	0.73, 1.72	15	13.3	**1.13**	0.63, 1.86	33	32.8	**1.00**	0.69, 1.41
Other sites	2499	2426.3	**1.03**	0.99, 1.07	857	815.0	**1.05**	0.98, 1.12	797	762.4	**1.05**	0.97, 1.12	845	848.9	**1.00**	0.93, 1.06
males	1391	1355.5	**1.03**	0.97, 1.08	485	462.8	**1.05**	0.96, 1.15	462	427.2	**1.08**	0.99, 1.18	444	465.5	**0.95**	0.87, 1.05
females	1108	1070.8	**1.03**	0.97, 1.10	372	352.2	**1.06**	0.95, 1.17	335	335.2	**1.00**	0.90, 1.11	401	383.4	**1.05**	0.95, 1.15

* p < 0.05; † p < 0.01; CI = confidence interval; E = expected number; O = observed number; SIR = standardized incidence ratio

Table 3 shows, that the total number of observed cancers was close to the expected number (SIR 1.02, 95% CI: 1.00, 1.03), in males (SIR 1.00, 95% CI: 0.97, 1.02) as well as in females (SIR 1.03, 95% CI: 1.01, 1.06). The observed number of cancers of the respiratory system (predominantly lung cancer) in females was increased (SIR 1.13, 95% CI: 1.03, 1.24), but the number in both sexes combined was statistically significantly decreased (SIR 0.94, 95% CI: 0.90, 0.99). This was caused by a relatively low incidence in males (SIR 0.89, 95% CI: 0.84, 0.94). A similar pattern was observed for cancer of head and neck (SIR females 1.25, 95% CI 1.04, 1.49; SIR males 0.92, 95% CI 0.78–1.07). The incidence was statistically significantly increased for hematological malignancies (SIR 1.12, 95% CI: 1.05, 1.19, 1044 cases). The raised risk was most prominent in males (SIR males 1.18, 95% CI: 1.09, 1.28, SIR females 1.04, 95% CI: 0.95, 1.14). A statistically significantly increased incidence was observed for NHL (SIR 1.22, 95% CI: 1.12, 1.33, 516 cases), while the confidence interval for acute lymphoblastic leukemia (ALL; SIR 1.34, 95% CI: 0.95, 1.83, 39 cases) included unity. A relatively low rate was observed for Hodgkin lymphoma (SIR 0.78, 95% CI: 0.58, 1.04).

Classification of lymphoid malignancies according to the WHO-classification, revealed relatively high rates for lymphoplasmocytic lymphoma (SIR 1.5, 95% CI: 1.1, 2.9), follicular lymphoma (SIR 1.5, 95% CI: 1.2, 1.8), diffuse large B-cell lymphoma (SIR 1.6, 95% CI: 1.4, 1.9) and T-cell lymphoma (SIR 1.4, 95% CI: 1.0, 1.8). The rates for plasma cell tumors (SIR 1.1, 95% CI 0.9, 1.3), small lymphocytic lymphoma/chronic lymphocytic leukemia (SIR 0.8, 95% CI: 0.6, 1.0) and other & unspecified lymphoma/leukemia (SIR 1.0, 95% CI: 0.8, 1.2) were not increased.

Cancer was diagnosed in 74 children up to 15 years of age, which was relatively high (SIR 1.26, 95% CI 0.99, 1.58), due to the higher than expected number of children with ALL (23 cases, SIR 1.59, 95% CI 1.01, 2.39).

For most cancer sites, the SIRs for the periods 1988–1993, 1994–1998 and 1999–2003 were quite similar. The increased risk of hematological malignancies was consistently observed in the three time periods. An increased number of breast cancer cases was observed in the 1994–1998 period (SIR 1.09, 95% CI 1.01, 1.18). An increased number of cancer of the bladder and other urinary organs in males was only observed in 1988–1993 (SIR 1.24, 95% CI 1.06, 1.44).

### Cancer incidence in the core zone

Table 4 shows that cancer incidence in the core zone was slightly increased in comparison to the national incidence (SIR 1.06, 95% CI 1.02, 1.10) as well as in comparison to the ring zone (RR 1.05, 95% CI 1.01, 1.10), mostly because of an increased incidence in males (SIR 1.07, 95% CI 1.01, 1.13; RR 1.09, 95% CI 1.03, 1.16). Statistically significantly increased numbers in the core zone in comparison to the ring zone were observed for cancer of the respiratory system (RR 1.27, 95% CI 1.12, 1.45) and prostate (RR 1.17, 95% CI 1.02, 1.34) in males and for cancer of the genital organs in females (RR 1.24, 95% CI 1.04, 1.50), based on increased RRs for each specific site (cervix 1.20, corpus 1.04, ovary 1.55, vulva & other 1.17). In comparison to the national incidence only cervical cancer and ovarian cancer were increased (SIR cervix 1.29, corpus 1.00, ovary 1.32, vulva & other 0.98). In the core zone, the incidence rate of bladder cancer in males (SIR 1.26, 95% CI 1.01, 1.56; RR 1.20, 95% CI 0.94, 1.52) was also relativity high. The incidence of hematological malignancies was higher in the core zone than in the to the ring zone, but the increase was not statistically significant (RR 1.06, 95% CI 0.91, 1.24).

**Table 4:** Number of cancer cases in subjects with residence in the Schiphol area according to site, gender and area of residence, 1988–2003

*cancer site (ICD-10 code) and gender*	*area of residence*							
	
	*ring zone*			*core zone*				
		
		*parameter*			*parameter*			
				
	*number of cases*	*SIR*^#^	*95% CI*	*number of cases*	*SIR*^#^	*95% CI*	*RR*^##^	*95% CI*
All malignancies (C00–C95)	10 855	1.01	0.99, 1.03	2 352	1.06*	1.02, 1.10	1.05*	1.01, 1.10
adult males	5 440	0.98	0.96, 1.01	1 257	1.07	1.01, 1.13	1.09*	1.03, 1.16
adult females	5 356	1.03	1.00, 1.06	1 080	1.04	0.98, 1.11	1.01	0.95, 1.08
children (<15)	59	1.25	0.95, 1.61	15	1.29	0.72, 2.13	1.04	0.59, 1.83
Head & neck (C00–C14)	238	1.06	0.93, 1.21	44	0.90	0.65, 1.21	0.85	0.61, 1.17
males	133	0.92	0.77, 1.09	29	0.89	0.59, 1.27	0.96	0.64, 1.44
females	105	1.32*	1.08, 1.59	15	0.93	0.52, 1.53	0.70	0.41, 1.21
Gastrointestinal tract (C15–C26)	2 422	0.99	0.95, 1.03	467	0.96	0.87, 1.05	0.97	0.88, 1.07
males	1 240	0.98	0.92, 1.03	254	0.95	0.83, 1.07	0.97	0.85, 1.11
females	1 182	1.00	0.95, 1.06	213	0.97	0.85, 1.11	0.97	0.84, 1.12
Respiratory system (C30–C34)	1 484	0.91*	0.86, 0.96	378	1.10	0.99, 1.22	1.21*	1.08, 1.35
males	1 085	0.85*	0.80, 0.90	293	1.08	0.96, 1.21	1.27*	1.12, 1.45
females	399	1.13*	1.02, 1.24	85	1.17	0.93, 1.44	1.04	0.82, 1.31
Breast (C50)	1 731	1.05*	1.01, 1.11	356	1.04	0.93, 1.15	0.99	0.88, 1.11
Female genital organs (C51–C58)	567	0.93	0.86, 1.01	143	1.16	0.98, 1.37	1.24*	1.04, 1.50
Prostate (C61)	1 044	1.02	0.96, 1.08	247	1.19*	1.05, 1.35	1.17*	1.02, 1.34
Bladder & other urinary tract (C65–C68)	468	1.09	0.99, 1.19	102	1.18	0.96, 1.43	1.16	0.93, 1.43
males	341	1.05	0.95, 1.17	84	1.26*	1.01, 1.56	1.20	0.94, 1.52
females	100	0.93	0.76, 1.13	18	0.92	0.54, 1.44	0.98	0.60, 1.63
Hematological malignancies (C81–C96)	855	1.10*	1.03, 1.18	189	1.17*	1.01, 1.35	1.06	0.91, 1.24
males	483	1.16*	1.06, 1.27	115	1.26*	1.04, 1.51	1.09	0.89, 1.33
females	372	1.04	0.94, 1.15	74	1.06	0.83, 1.33	1.02	0.79, 1.31
Other sites	2 073	1.03	0.99, 1.08	426	1.02	0.93, 1.12	0.99	0.89, 1.10
males	1 148	1.03	0.97, 1.09	243	1.01	0.89, 1.14	0.98	0.85, 1.12
females	925	1.03	0.97, 1.10	183	1.04	0.90, 1.20	1.01	0.86, 1.18

* p < 0.05

^# ^reference population: the Netherlands 1989–2003

^## ^ratio of the SIR of the core zone and the SIR of the ring zone

CI = confidence interval; SIR = standardized incidence ratio; RR = rate ratio

## Discussion

The major finding of our study is that total cancer incidence in the area around Schiphol airport was almost equal to the national cancer incidence (SIR 1.02). Furthermore, the incidence of hematological malignancies was statistically significantly increased, while the incidence of cancer of the respiratory system was statistically significantly decreased. We observed an excess risk in children aged 0–14 (SIR 1.26). The cancer incidence in the core zone was slightly increased in comparison to the ring zone, due to an excess risk of cancer of the respiratory tract and prostate in males and cancer of the genital organs in females.

As the overall incidence of cancer of the respiratory tract was decreased (SIR 0.94), this observation does not support a positive association between the airport and the occurrence of cancer of the respiratory tract. The incidence pattern of respiratory system cancer in the Schiphol area, i.e. low rates in males and somewhat higher rates in the core zone and among females, is well within the normal regional variation in the Netherlands. Because smoking is the most important risk factor for lung cancer [15], and there is evidence of substantial regional variation in smoking habits in the Netherlands [16], smoking is likely to be responsible for the differences in respiratory system cancer (mainly lung cancer) between the Schiphol area and the Netherlands overall. Unfortunately, no data on smoking habits according to postal code in the Schiphol area are available. Lung cancer incidence in the 1990s in males was lowest in high income areas in the Netherlands. In females, low rates were found in rural areas, while high rates were observed in urban areas [17]. The slightly increased incidence of cancer of the respiratory system in females is in accordance with the moderately urbanized status of the Schiphol area. The data on per capita income (table 2) support the assumption that the low incidence of cancer of the respiratory system in males is related to the high per capita income of the Schiphol area. However, within the Schiphol area only a weak association was observed between the incidence of lung cancer and per capita income by postal code area (data not shown). This may be due to relatively small numbers by postal code area and the long induction period of lung cancer as the regional variation in lung cancer incidence can best be explained by the smoking habits 10 to 30 years ago.

In a number of studies in urban areas an increase of lung cancer incidence or mortality was observed [18,19], mostly attributed to differences in smoking habits. However, there is increasing evidence for a relation between lung cancer risk and ambient air pollution [20,21]. Although we cannot exclude the possibility that the incidence of cancer of the respiratory system in the absence of the airport would even have been lower than the observed incidence, the pattern of the observed incidence does not render this very likely.

The statistically significantly increased rate for breast cancer in 1994–1998 (SIR 1.09 for the total study area) is also within the observed regional variation in the Netherlands. Part of this variation can be explained by local variation in the start of the national screening program for breast cancer. The relatively high incidence of breast cancer in 1994–1998 is probably related to the start of screening in the Schiphol area in that period.

We do not have an explanation for the relatively high incidence of cancer of the female genital organs in the core zone (RR in comparison to the ring zone 1.24). Possibly, this is only a chance finding, as despite the large variation in risk factors for the specific sites the incidence of all specific sites was increased, while the SIR in comparison to the general population was not statistically significantly increased. An association with pollution has not been described for cancer of the female genital organs. Moreover, in the total study area the incidence of these cancers was not increased (SIR 0.97).

The most striking observation in our study is the increased incidence of hematological malignancies, which was observed consistently over three periods, mostly in males but also in females. The increase was more pronounced in the core zone. The increased incidence was mostly due to increased numbers of cases of ALL and NHL (especially lymphoplasmocytic lymphoma, follicular lymphoma, diffuse large B-cell lymphoma and T-cell lymphoma, but not small lymphocytic lymphoma/chronic lymphocytic leukemia [SLL/CLL]), while the incidence of Hodgkin lymphoma was decreased. However, pathology could not be reviewed in this study and different classification systems of lymphoma have been used by pathologists during the study period.

The moderately increased cancer incidence in children (about one supplementary case per year) was mainly caused by the increased number of cases of ALL, as ALL occurs mainly in children.

Also from a national perspective, the number of cases of NHL was markedly increased. In the Netherlands in 1989–1998, the highest rate of NHL in males was found in Greater-Amsterdam, which includes the Schiphol area (source: the Netherlands Cancer Registry). In females in Greater-Amsterdam, the incidence of NHL was also relatively high. Chance is not a likely explanation for our finding of an increased incidence of NHL, since the increase was consistently observed over three time periods. The relatively high incidence of NHL in the Schiphol area is also consistent with an increased mortality due to NHL already reported in Haarlemmermeer in 1981–1986 [7]. In several studies, an increased risk of hematological malignancies was found for farmers, which is related to the use of pesticides, infectious micro-organisms or working with beef cattle [22-27]. However, although the Schiphol area includes a few areas with intensive agricultural activities, the increased risk for hematological malignancies was also found in areas with few agricultural activities.

Several studies have shown that the incidence of NHL is correlated with nitrate in municipal drinking water due to nitrogen fertilizers [28,29] and is increased in urban/industrialized areas [30,31]. Hatzissabas *et al *found that the incidence of large cell high malignancy lymphomas is highest in industrialized regions with pollution of water supplies by more toxic and immunosuppressive substances, while CLL is more frequent in areas with rather low-dose chronic influences such as from the use of fertilizers and pesticides in farming [32]. The pattern of NHL in the Schiphol area – increase of follicular and diffuse large B-cell lymphoma, but not SLL/CLL – might indicate a relation with pollution which is also found in urban areas.

However, an association between the incidence of hematological malignancies and the environment in the Schiphol area is not supported by the available data on ambient air quality. Measurements in 1989 at the airport grounds of Schiphol showed increased levels of ambient air pollutants, including polycyclic aromatic hydrocarbons which are probably or possibly carcinogenic according to the International Agency for Research on Cancer, but not in the direct vicinity outside the airport ground [33]. Morphology and composition of soot emitted by aircraft at Schiphol showed great similarities with soot emitted by the road traffic. Only different profiles of hydrocarbons in the range of C_6_–C_12 _in emissions from aircraft engines, aviation fuels and road traffic were reported. Since 1994, three locations in the vicinity of Schiphol are part of the provincial monitoring network for ambient air quality measurement [34]. During 1994–2002, the concentrations of the air pollutants NO_2_, CO, O_3_, PM_10 _(particulate matter <10 μm), benzo(a)pyrene, benzene and black smoke at the three locations in the Schiphol area were stable and well comparable to urban background levels in Amsterdam [9]. A more detailed investigation at 59 additional locations in the Schiphol area in 2000/2001 revealed that the average contribution of air traffic emissions and of aviation fuel storage and transfer to the total concentration of volatile hydrocarbons in the area around Schiphol were only 3%, and up to 5–7% at individual locations [35]. Road traffic contributed 28%. For CO, NO_2 _and PM_10_, no relevant influence of emissions of Schiphol on ambient pollutant levels could be determined. Although we cannot exclude the possibility that residents of the Schiphol area have been exposed to air pollutants that were not measured or that higher levels of air pollutants have existed in the past, the results of the ambient air quality monitoring and the source appointment of air pollutants render it unlikely that aircraft emissions have contributed substantially to the total levels of pollutants in the ambient air of the Schiphol area. It therefore seems unlikely that the increased incidence of hematological malignancies is specifically related to ambient air pollution caused by aircraft emissions.

Our results should be interpreted considering the strengths and limitations of the study design. An advantage is the availability of high quality data from a population-based cancer registry over a period of sixteen years. However, the use of the national cancer incidence as a reference has its limitations. Preferably, the cancer incidence in a population which is comparable to the Schiphol region as far as urbanization, socio-economic status and smoking habits, should be used. Unfortunately, such a reference population is not available. Another limitation of the study is that only cancer cases that were residents of the Schiphol area at the date of diagnosis were included in the study. Part of the original residents will have left the area, while others only recently settled in the area. The effect of migration (non-differential misclassification) usually results in an underestimation of the risk at study.

## Conclusion

The overall cancer incidence in the Schiphol area was similar to the national incidence in the Netherlands. An association was found between residence in the Schiphol area and a moderately increased incidence of hematological malignancies, especially NHL and ALL. However, the increased risk of hematological malignancies could not be explained by higher levels of ambient air pollution in the Schiphol area, while similarly increased rates were observed in Greater Amsterdam. Further studies, for example a study with focus on substances in urban ambient air pollution, are necessary in order to elucidate the causes of the observed association.

## Competing interests

The author(s) declare that they have no competing interests.

## Authors' contributions

JvW and FvL conceived the study and were involved in the design. OV performed the statistical analysis and wrote the first draft of the article. All authors were involved in the interpretation of the results and the revision of the draft. All authors read and approved the final manuscript.

## Pre-publication history

The pre-publication history for this paper can be accessed here:



## References

[B1] Tesseraux I (2004). Risk factors of jet fuel combustion products. Toxicol Lett.

[B2] Passchier W, Knottnerus A, Albering H, Walda I (2000). Public health impact of large airports. Rev Environ Health.

[B3] Knipschild PG (1976). Medische gevolgen van vliegtuiglawaai (Health effects of aircraft noise). Thesis.

[B4] Rosenlund M, Berglind N, Pershagen G, Jarup L, Bluhm G (2001). Increased prevalence of hypertension in a population exposed to aircraft noise. Occup Environ Med.

[B5] Heisterkamp SH, Doornbos G, Nagelkerke NJ (2000). Assessing health impact of environmental pollution sources using space-time models. Stat Med.

[B6] Haines MM, Stansfeld SA, Job RF, Berglund B, Head J (2001). A follow-up study of effects of chronic aircraft noise exposure on child stress responses and cognition. Int J Epidemiol.

[B7] Van Bruggen M, Van Wijnen JH (1989). De kankersterfte in de gemeente Haarlemmermeer (1981–1986). Een oriënterend descriptief onderzoek. (Cancer mortality in Haarlemmermeer; a descriptive study).

[B8] Visser O, Van Wijnen JH, Benraadt J, Van Leeuwen FE (1997). Incidentie van kanker in de omgeving van Schiphol in 1988–1993.(Incidence of cancer in the vicinity of Schiphol airport, 1988–1993). Ned Tijdschr Geneeskd.

[B9] Meijer W, De Jonge D (2003). Datarapport Luchtkwaliteit Haarlemmermeer. Resultaten 2002. (Air quality Haarlemmermeer; results 2002).

[B10] Staatsen BAM, Franssen EAM, Doornbos G, Abbink F, Van der Veen AA, Heisterkamp SH, Lebret E (1993). Gezondheidskundige evaluatie Schiphol. (Health evaluation Schiphol).

[B11] Van der Sanden GA, Coebergh JWW, Schouten LJ, Visser O, Van Leeuwen FE (1995). Cancer Incidence in the Netherlands in 1989 and 1990. First results of the nationwide Netherlands Cancer Registry. Eur J Cancer.

[B12] Visser O, Coebergh JWW, Schouten LJ (1996). Incidence of cancer in the Netherlands 1993 Fifth report of the Netherlands Cancer Registry.

[B13] Visser O, Van Dijck JAAM, Siesling S (2003). Incidence of cancer in the Netherlands 1999/2000 Eleventh report of the Netherlands Cancer Registry.

[B14] Altman DG (1991). Practical statistics for medical research.

[B15] Doll R, Peto R (1979). Mortality in relation to smoking: 20 years' observation on male British doctors. Br Med J.

[B16] Mackenbach JP, Kunst AE, Looman CWN, Van Beeck EF (1992). Regionale sterfteverschillen in Nederland. (Regional mortality patterns in the Netherlands). T Soc Gezondheidsz.

[B17] Van Dijck JAAM, Coebergh JWW, Siesling S, Visser O (2002). Trends of cancer in the Netherlands, 1989–1998.

[B18] Doll R (1978). Atmospheric pollution and lung cancer. Environ Health Perspect.

[B19] Hoogendoorn D (1983). Regionale verschillen in kankersterfte. (Regional cancer mortality patterns). Ned T Geneesk.

[B20] Cohen AJ, Pope CA (1995). Lung cancer and air pollution. Environ Health Perspect.

[B21] Vineis P, Forastiere F, Hoek G, Lipsett M (2004). Outdoor air pollution and lung cancer: recent epidemiologic evidence. Int J Cancer.

[B22] Khuder SA, Mutgi AB, Schaub EA, Tano BD (1999). Meta-analysis of Hodgkin disease among farmers. Scand J Work Environ Health.

[B23] Khuder SA, Schaub EA, Keller-Byrne JE (1998). Meta-analyses of non-Hodgkin lymphoma and farming. Scand J Work Environ Health.

[B24] Viel JF, Richardson ST (1993). Lymphoma, multiple myeloma and leukaemia among French farmers in relation tot pesticide exposure. Soc Sci Med.

[B25] Amadori D, Nanni O, Falcini F (1995). Chronic lymphocytic leukaemias and non-Hogdkin's lymphomas by histological type in farming-animal breeding workers: a population case-control study based on job titles. Occup Environ Med.

[B26] Keller JE, Howe HL (1994). Case-control studies of cancer in Illinois farmers using data from the Illinois State Cancer Registry and the U.S. Census of Agriculture. Eur J Cancer.

[B27] Fritschi L, Johnson KC, Kliewer EV, Fry R, Canadian Cancer Registries Epidemiology Research Group (2002). Animal-related occupations and the risk of leukemia, myeloma, and non-Hodgkin lymphoma in Canada. Cancer Causes Control.

[B28] Gulis G, Czompolyova M, Cerhan JR (2002). An ecologic study of nitrate in municipal drinking water and cancer incidence in Trnava District, Slovakia. Environ Res.

[B29] Weisenburger DD (1990). Environmental epidemiology of non-Hodgkin lymphoma in eastern Nebraska. Am J Ind Med.

[B30] Schouten LJ, Meijer H, Huveneers JAM, Kiemeney LALM (1996). Urban-rural differences in cancer incidence in the Netherlands, 1989–1991. Int J Epidemiol.

[B31] Doll R (1991). Urban and rural factors in the aetiology of cancer. Int J Cancer.

[B32] Hatzissabas I, Krueger GR, Medina JR, Bedoya VA, Papadakis T (1993). Environmental pollution and malignant lymphomas: a tentative contribution to geographic pathology. Anticancer Res.

[B33] Van den Anker IM, Van Velze K, Onderlinden D (1989). Luchtverontreiniging door de luchthaven Schiphol (Air pollution by Schiphol airport).

[B34] Nieuwenhuis JW, Van der Meij M, Lucas MPA, De Jonge D (1995). Luchtkwaliteit Haarlemmermeer 1994 (Air quality Haarlemmermeer).

[B35] Thijsse TR, Van Loon M (2001). Nader onderzoek naar de luchtkwaliteit in de omgeving van Schiphol en de bijdrage van te onderscheiden bronnen. (Air quality in the Schiphol area; contribution of different sources).

